# Pediatric liver failure: we came, we saw, but have we conquered?

**DOI:** 10.12688/f1000research.9623.1

**Published:** 2017-08-22

**Authors:** Sara Kathryn Smith, Philip Rosenthal

**Affiliations:** 1 Division of Pediatric Gastroenterology, Hepatology, and Nutrition, University of California, San Francisco, Box 0136, San Francisco, CA, 94143, USA

**Keywords:** pediatric acute liver failure, scoring systems, diagnosis, liver transplantation

## Abstract

Although there have been advances made in the diagnosis and management of pediatric acute liver failure, there is still no consensus regarding the definition or standardized evaluation, and an inability to predict outcomes, specifically irreversible brain injury, in many patients exists. Much of the research surrounding pediatric acute liver failure in the last several years has centered on the development of predictive scoring systems to enhance diagnosis and treatment decisions. In this article, we will discuss our current understanding of liver failure and updated management strategies in children with acute liver failure.

Advances continue to be made in the diagnosis and management of pediatric acute liver failure (PALF); however, there is still no consensus regarding standardized evaluation, and an inability to predict outcomes, specifically irreversible brain injury, in many patients exists. PALF is a rapidly evolving condition with different etiologies, management strategies, and outcomes when compared to acute liver failure (ALF) in adults. As with many pediatric disease processes, much of our knowledge surrounding the management of PALF has been extrapolated from experiences with adults. The clinical study of PALF is limited by the heterogeneity of underlying etiology, the small number of cases, and the difficulty in predicting outcomes. While there is no firm definition for ALF in children, entry criteria in the PALF longitudinal research study included 1) absence of known, chronic liver disease, 2) liver-based coagulopathy not responsive to parenteral vitamin K, and 3) international normalized ratio (INR) between 1.5 and 1.9 in the presence of clinical evidence of encephalopathy or INR 2.0 and higher without clinical signs of encephalopathy ^1^. The various etiologies of ALF in children show some correlation with age groups, and it is important to determine when PALF is secondary to a potentially treatable cause, such as herpes simplex virus, gestational alloimmune liver disease, autoimmune hepatitis (AIH), acute acetaminophen (APAP) toxicity, or Wilson’s disease.

PALF can present in various ways but common features at presentation, based on data from a national registry, include encephalopathy, seizure, and ascites ^1^. When PALF is recognized, it is imperative to determine if the patient has a condition that is potentially treatable and if a liver transplant is necessary and appropriate for patient survival. Unlike in adults, up to 50% of cases of PALF are deemed indeterminate, and these children are more likely to undergo liver transplant. Prognosis in PALF is affected by the development of complications, including cerebral edema, overwhelming sepsis, and multi organ failure, including renal failure. At this time, we still do not have reliable tools to predict survival or death in PALF. Combinations using biochemical tests, clinical features, and etiology have been utilized but none have proven to be reliable. Existing predictive scoring systems, such as the Kings College Hospital Criteria (KCHC), were developed prior to liver transplant when patient outcomes were limited ^2^. Use of the KCHC in a cohort of PALF patients showed a positive predictive value of only 33% (compared to 97% in the original study), raising concerns for over-utilization of liver transplant in PALF ^3^. The Pediatric End Stage Liver Disease (PELD) score was developed specifically for pediatric patients; however, its application is limited to chronic rather than acute liver disease, with inclusion of growth failure, which is irrelevant to prognosis in PALF. An additional score, named the Liver Injury Unit Scoring System, has been shown to be predictive of survival without liver transplant in PALF. This scoring system includes factors for peak total bilirubin, prothrombin time or INR, and ammonia ^4^.

In general, the management of PALF includes an attempt to determine etiology, guided by the patient’s age with prioritization of treatable diagnoses, monitoring of individual organ systems, identification and treatment of complications, and supportive care. One of the limiting factors to studying PALF is the lack of standardization for evaluation and the frequent incomplete work up of patients because of clinical improvement, limitations to obtaining timely lab testing, loss to follow up, or death. Narkewicz *et al*. examined the patterns of diagnostic evaluation in PALF and found that many patients with an indeterminate PALF diagnosis were not screened for the four leading causes of ALF (AIH, drug exposure, hepatitis A, and fatty acid oxidation defects) ^5^. Data from the same study suggest that there is room for improvement in the diagnosis of metabolic liver disease and AIH in children presenting with ALF. The inability to determine a diagnosis can lead to missed opportunities for potential treatments, and establishing a clear diagnosis can impact the decision to move forward with liver transplant or to defer liver transplant in cases of poor prognosis, such as genetic mitochondrial defect disorders.

Although some PALF patients recover without transplantation, mortality remains high as a result of sepsis, cerebral edema, and multi organ failure. Hepatic encephalopathy (HE) in children can be subtle, making it difficult to recognize, and may not be present until the end stages of liver failure. Ng and colleagues investigated the outcomes of children with and without HE and found that mortality 21 days after enrollment was highest in patients with severe HE (grades III or IV) or in patients demonstrating HE progression, with only 25% of patients with grade III or IV HE showing spontaneous recovery ^6^. Importantly, their data demonstrated that children with PALF without overt HE are still at risk for death. It is important to attempt to establish cases in which the severity of HE predicts unfavorable neurological progression, despite transplantation. Patients should be evaluated for hypoglycemia, infection, intracranial hemorrhage, and drug intoxication with any sudden changes in mental status. Electroencephalography can show significant alterations but is not useful for identifying intracranial hypertension due to progressive liver failure. Transcranial Doppler ultrasonography is a noninvasive method of measuring cerebral blood flow velocity, which can be used to monitor changes in cerebral hemodynamics and can be utilized to determine patients in whom transplantation is contraindicated. Intracranial pressure (ICP) monitoring is another method for measuring variation in ICP and can help to guide management; however, its use is controversial owing to its invasive nature and the high risk due to the presence of moderate to severe coagulopathy that accompanies ALF. The incidence of intracranial hemorrhage in ALF is less than 1%, and when it does occur it is often associated with ICP monitoring ^7^. A retrospective review of PALF in the United States from 2008 to 2013, including 583 patients with PALF admitted to 16 liver transplant centers, showed decreasing frequency of the use of invasive ICP monitoring and no association with improved survival in patients with cerebral edema ^8^.

Traditionally, continuous renal replacement therapy (CRRT) has been used to manage renal dysfunction, which is a common complication of PALF, and it has been shown to successfully reduce ammonia, reduce lactate, and optimize fluid balance in adult patients. Deep and colleagues investigated the use of CRRT in PALF and found that its early institution can help prevent further deterioration and lead to spontaneous recovery or, in more severe cases, can help bridge patient to liver transplant ^9^. A total of 165 pediatric patients admitted with ALF from January 2003 to December 2013 were studied. Of these patients, 45 received CRRT prior to transplantation and/or recovery, with indications being hyperammonemia >200 μmol/L or HE lower than grade 2. Of the patients with PALF who did not undergo liver transplant, those who received CRRT had a significantly increased chance of survival. A decrease in ammonia by 48 hours after the initiation of CRRT significantly improved survival, and for every 1-hour delay in the initiation of CRRT, the likelihood of mortality increased by 4%.

The use of therapeutic plasmapheresis in ALF has been associated with higher mortality, lower probability of survival with native liver, and increased complications, including acute kidney injury, sepsis, acute respiratory failure, cardiovascular compromise, and HE ^10^. There continues to be discussion regarding the utility of liver biopsy in PALF, especially in the presence of significant coagulopathy. Some studies have suggested that liver biopsy in PALF may help in establishing the diagnosis, investigating the immune response, and assessing the degree of liver necrosis.

Several temporizing artificial support systems which remove toxins from the circulation have been developed recently. Biological extracorporeal liver assist devices utilize living hepatocytes to filter blood or plasma from the patient. Hepatocytes may be porcine or human in origin ^11^. MARS (Molecular Absorbent Recirculating System; Flux 2.1) and Prometheus (Fresenius Medical Care AG, Bad Homburg, Germany) are non-biological extracorporeal assist devices based on hemadsorption. Currently, these systems have not demonstrated clear effectiveness or survival benefit in ALF ^12^.

A study using data from the PALF study group found that autoantibodies occur in 28% of children presenting with ALF, with increased frequency in children with a final diagnosis of AIH ^10^. Autoantibodies were not associated with 21-day outcomes in children presenting with ALF. Autoantibody presence in children with ALF does not preclude the need for complete diagnostic evaluation because these antibodies have been found in various conditions. The significance of autoantibodies in children with ALF still remains unclear; however, the presence of positive anti-liver kidney microsomal antibodies may represent a unique population of children and requires further study.

N-acetylcysteine (NAC) is known to improve prognosis in APAP-induced ALF ^13^, and the use of NAC has also been shown to improve transplantation-free survival in adults with non-APAP ALF and grade 1–2 HE ^14^. Patients in the PALF study group were allocated to receive either a continuous intravenous infusion of NAC or placebo for up to 7 days. One-year survival was the primary outcome with liver transplantation-free survival, liver transplantation, length of intensive care unit and hospital stays, organ system failure, and maximum HE score as secondary outcomes ^15^. NAC did not improve 1-year survival in non-APAP PALF and, in fact, lowered 1-year liver transplant-free survival, particularly in children under 2 years of age.

The use of steroids in PALF needs additional investigation. A recent study using patients in the PALF study group showed that steroid treatment was not significantly associated with improved 21-day survival in patients with positive autoantibodies. In fact, there was the suggestion that steroids may be dangerous in some cases of PALF, with more deaths in patients with positive autoantibodies in diagnoses other than AIH or other immune dysregulation syndromes ^10^.

Despite changes in management and improvements in the diagnosis of PALF and emphasis on timely referral to a liver transplant medical center, many patients will undergo liver transplantation. While liver transplant has improved short-term survival in PALF, long-term survival remains poor compared with other indications for liver transplantation. The 6-month probability of survival post-transplant in children with ALF is estimated to be 75.9% compared to 90.8% for children transplanted for other diagnoses ^16^. Decisions to move forward with liver transplant must include consideration of the risks of surgery and the critical shortage of donor organs. Increasing numbers of transplant centers are now utilizing living donor liver transplants with good outcomes. Living donor transplants offer the advantage of optimization of the timing of transplant, shorter cold ischemia times, and improved quality organs ^17^. Auxiliary liver transplantation may be considered in a small number of patients who fulfill the criteria for liver transplantation, with one study demonstrating survival rates of 85% at 1, 5, and 10 years ^18^. It is vital that patients presenting with PALF are transferred early in their clinical course to an experienced pediatric liver transplant center, even in the absence of severe coagulopathy and/or encephalopathy.

While our understanding of the diagnosis and management of PALF continues to improve, it remains a difficult and challenging entity. We have still not developed a standardized approach to diagnosis and management, largely because of the variation in clinical presentation. It will be important to develop a method to reliably distinguish those patients who require liver transplant for survival as well as determine when to initiate known methods of management for the sequelae of liver failure that ultimately contribute to mortality. Future research should include the analysis of center-specific protocols with a focus on the influence of provider decision making and region organ availability in the selection of patients for liver transplant.

## Abbreviations

AIH, autoimmune hepatitis; ALF, acute liver failure; APAP, acetaminophen; CRRT, continuous renal replacement therapy; HE, hepatic encephalopathy; ICP, intracranial pressure; INR, international normalized ratio; KCHC, Kings College Hospital Criteria; NAC, N-acetylcysteine; PALF, pediatric acute liver failure.
